# The Role of Glucagon-Like Peptide-1 Agonists in the Treatment of Multiple Sclerosis: A Narrative Review

**DOI:** 10.7759/cureus.67232

**Published:** 2024-08-19

**Authors:** Alan D Kaye, Kelly R Sala, Brennan M Abbott, Alexandra N Dicke, Landyn D Johnson, Parker A Wilson, Sam N Amarasinghe, Naina Singh, Shahab Ahmadzadeh, Adam M Kaye, Sahar Shekoohi, Giustino Varrassi

**Affiliations:** 1 Department of Anesthesiology, Louisiana State University Health Sciences Center, Shreveport, USA; 2 School of Medicine, Louisiana State University Health New Orleans, New Orleans, USA; 3 School of Medicine, Louisiana State University Health Sciences Center, Shreveport, USA; 4 Department of Pharmacy Practice, Thomas J. Long School of Pharmacy and Health Sciences, University of the Pacific, Stockton, USA; 5 Department of Pain Medicine, Paolo Procacci Foundation, Rome, ITA

**Keywords:** obesity, metformin, ozempic, liraglutide, diabetes mellitus, alzheimer’s, neurodegenerative disease, multiple sclerosis, glucagon-like peptide-1 agonists

## Abstract

Multiple sclerosis (MS) is a chronic, progressive autoimmune disease modulated by autoantibodies that inflame and destroy the myelin sheath encasing neuronal axons, impairing proper axonal conduction and function. Glucagon-like peptide-1 (GLP-1) receptor agonists have been demonstrated to exert anti-inflammatory and neuroprotective effects, making these drugs particularly exciting prospects in the treatment of MS. While the exact mechanism remains unclear, GLP-1 receptor agonists may modulate inflammatory responses by targeting GLP-1 receptors present on immune cells such as macrophages, monocytes, and lymphocytes. In animal models, GLP-1 agonists have been shown to significantly delay the onset and severity of experimental autoimmune encephalopathy symptoms, as well as to increase nerve myelination and brain weight. In further experiments using animal models of nerve crush injury, specimens given GLP-1 agonists reported a significant increase in the rate and density of nerve regeneration compared to controls. Thus, GLP-1 agonists show promise as both prophylactic and symptomatic treatment for MS and may provide further utility in the treatment of other autoimmune, inflammatory, and neurodegenerative conditions.

## Introduction and background

Multiple sclerosis (MS) is a chronic, progressive autoimmune disease affecting the central nervous system (CNS). Autoantibodies target the myelin sheaths, leading to inflammation, demyelination, and later axon damage [1]. The disruption of the myelin sheath impairs nerve signal transmission, manifesting in a variety of neurological symptoms such as paralysis, sensory disturbances, and visual impairment [2]. The most well-established hypothesis for how MS causes dysfunction is a failure of T cell tolerance mechanisms, which could be a failure of central tolerance in the thymus or peripheral tolerance in peripheral lymphoid tissues. This theory is supported by evidence that myelin antigen-specific T cells can be isolated from the peripheral blood lymphocytes of humans with MS [3]. These aberrant T cells activate B cells and other antigen-presenting cells, which cross the blood-brain barrier, leading to inflammation and demyelination of CNS myelin by pro-inflammatory cytokines and myelin-specific antibody production [4]. Damage to myelin causes nerve conduction block, disrupting the flow of electrical signals along nerve fibers. This disruption leads to the clinical manifestations of the disease. A relapsing and remitting pattern of symptoms is common in the early stages, with relapses mainly related to acute episodes of inflammatory demyelination, which decreases conduction across axons [2]. Recovery from relapse is associated with reduced inflammation, axon remyelination, and conduction restoration, decreasing the neurological symptoms and leading to remission. Approximately 60-70% of patients with relapsing-remitting MS eventually develop the progressive form, characterized by progressive neurological decline [5].

MS predominantly affects young adults and has a higher prevalence among women. Symptoms typically begin between 20 and 40 years of age. About 2.3 million people have MS globally, with a higher prevalence in Western countries and high-latitude countries [6]. The incidence of MS has been increasing since the 1950s, and the female-to-male ratio rose from 2:1 in the 1950s to approximately 3:1 in the 2010s, suggesting risk factors that predominantly affect women [6]. MS is the leading cause of disability among young adults and has a large economic burden. One study estimated the economic burden of MS in the United States to be $85 billion in 2019, projected to increase to $108 billion by 2039 [7]. Another study found that patients' out-of-pocket spending on just disease-modifying therapies (DMTs) for MS increased from $18.9 million in 2006 to $149.4 million in 2016 [8]. Additionally, people with MS experience significant work loss, working 9 hours less per week on average [7]. The progressive nature, high prevalence, and large economic burden of the disease provide a strong incentive to develop effective treatments for MS. Current treatments aim to manage relapses, slow disease activity, and treat symptoms. The primary treatment for acute relapses is corticosteroids, which reduce inflammation by decreasing immune system activity. They have been shown to alleviate symptoms and accelerate recovery from acute attacks, though they do not alter the course of the disease or the degree of recovery from an MS attack [9-10]. DMTs are a broad class of medications that alter the course of MS and are used for long-term treatment. Examples of these therapies include interferons, glatiramer acetate, and monoclonal antibodies. These therapies target specific aspects of the immune-mediated disease process, focusing on reducing early disease activity that is hypothesized to contribute to long-term disability [11]. The problem with DMTs is that they vary in efficacy, tolerability, and safety from patient to patient and also cost tens of thousands of dollars per year [12]. The variable effectiveness and high cost can negatively affect patient adherence to these medications. Glucagon-like peptide-1 (GLP-1) is a hormone released after eating that lowers glucose concentration by stimulating insulin secretion and suppressing glucagon release. GLP-1 also reduces gastric emptying and suppresses appetite [13]. GLP-1 receptor agonists (GLP-1 RAs) are a class of medications that mimic the actions of endogenous GLP-1. The FDA has approved several GLP-1 RAs, including exenatide, liraglutide, albiglutide, dulaglutide, lixisenatide, semaglutide, and tirzepatide, separated into short-acting and long-acting formulations. Short-acting agents delay gastric emptying, which lowers postprandial glucose, while long-acting agents lower fasting glucose levels [14]. Originally developed to treat type 2 diabetes, these drugs have proven to improve blood sugar control and promote weight loss without the hypoglycemic risk of other diabetes medications [15]. The FDA has approved seven GLP-1 RAs to improve blood sugar control and two for chronic weight management. Their unique effects make them a valuable treatment for these patients. While the exact mechanism remains unclear, GLP-1 receptor agonists may also play an important role in the modulation of inflammatory responses by targeting GLP-1 receptors in immune cells such as macrophages, monocytes, and lymphocytes.

**Figure 1:**
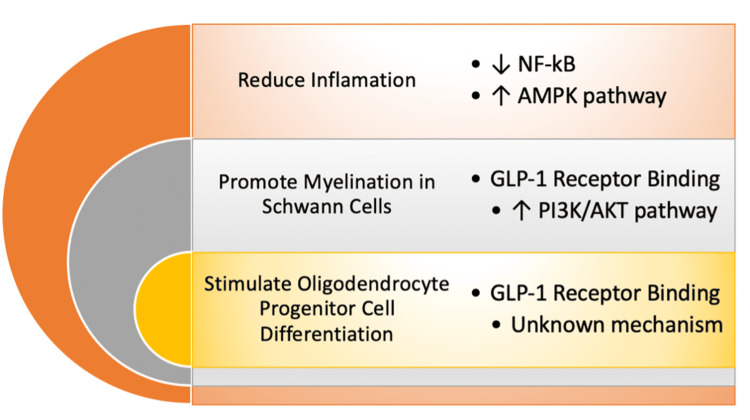
Summary of mechanisms by which GLP-1 agonists offer promising treatment potential in the disease pathogenesis and progression of multiple sclerosis. GLP-1: Glucagon-like peptide-1; PI3K/AKT: Phosphatidylinositol 3-kinase/protein kinase B; NF-kB: Nuclear factor-kB. Image credits: Kelly R. Sala and Alexandra N. Dicke.

GLP-1 RAs have also been shown to exert anti-inflammatory and neuroprotective effects. While the exact mechanism is not fully understood, GLP-1 RAs have been shown to reduce the activation of nuclear factor-kB (NF-kB) and increase the activation of the AMP-activated protein kinase (AMPK) pathway, both of which help suppress inflammation [16]. Both myelin-forming Schwann cells and oligodendrocytes have been found to express the GLP-1 receptor [17]. Stimulation of the GLP-1 receptor activates the phosphatidylinositol 3-kinase/protein kinase B (PI3K/AKT) pathway, which is crucial for initiating myelination in Schwann cells [18]. GLP-1 RAs have also shown the ability to stimulate oligodendrocyte progenitor cell differentiation (Figure 1) [17].

MS is a chronic autoimmune disorder with significant individual and societal impacts. The disease's high prevalence and increasing economic burden provide a strong incentive for developing effective treatments. Corticosteroids address the inflammatory symptoms of MS but not the underlying pathophysiological process. DMTs target the underlying process but come with variable efficacy and are cost-prohibitive. GLP-1 RAs could offer a novel therapeutic approach for treating MS. Although specific studies on their effectiveness in MS are limited, their anti-inflammatory and neuroprotective effects could play a crucial role in managing the disease. This review focuses on exploring GLP-1 RAs as a promising and cost-effective addition to the treatment of MS.

## Review

Clinical studies

Clinical studies on GLP-1 agonists and multiple sclerosis investigations have utilized various research methods and models. One study explored the therapeutic potential of NLY01, a novel GLP-1 agonist, in a mouse model of MS, experimental autoimmune encephalopathy (EAE) [19]. Using a sample of 51 mice, 25 were given NLY01 (10 mg/kg) twice weekly, while 26 received saline. The study showed that administration of NLY01 at the same time as EAE induction significantly delayed symptom onset and decreased symptom severity (p ≤ 0.0001). The effects of liraglutide were investigated on EAE in female C57 black 6 (C57BL/6) mice [20]. The study had a sample size of 13-28 mice per group. The experimental group received 10 μg/kg/day of liraglutide starting eight days post-immunization. The group receiving liraglutide experienced delayed disease score and onset of EAE compared to the control (EAE vs. EAE + Lira 14.31 ± 2.51 dpi vs. 17.44 ± 4.13 dpi, p = 0.031). Semaglutide was studied on EAE in male Swiss-Albino mice [21]. A total of 40 mice were utilized, with 20 mice induced with EAE receiving 25 nmol/kg/day i.p. of semaglutide 2 hours post EAE induction and 10 mice receiving 0.2 mL i.p. saline vehicle. The treatments continued for two weeks. The study found that semaglutide treatment reduced the EAE clinical score compared to the EAE control mice from a median score of 3 to a median score of 1 (p < 0.05). The mice receiving semaglutide also had a 60.88% increase in myelinated nerve fibers compared to the EAE control group (p < 0.05).

Another investigation focused on the prophylactic effects of dulaglutide in an EAE mice model [22]. The study used a sample size of 29 C57BL/6 mice. In the prophylactic group, 12 mice received dulaglutide on days 0, 3, 7, and 10 post-immunization. In the control group, eight mice received a saline vehicle. The prophylactic dulaglutide treatments decreased the clinical score of EAE symptoms from 3.781 ± 0.2083 to 2.021 ± 0.3053 (p < 0.001). The same study also examined a semi-therapeutic dose of dulaglutide administered to 9 mice on days 9, 12, 16, 19, 23, and 26 post-immunizations. The clinical score of the semi-therapeutic dose was also lower than the vehicle group (3.781 ± 0.2083 to 2.889 ± 0.2887; p < 0.05). The effects of liraglutide were evaluated on EAE-induced female Lewis rats [23]. The investigators started with a sample size of 30 rats, and the study ran for 11 days. Fifteen rats were given twice-daily liraglutide (200 μg/kg s.c.), and 15 were given a saline vehicle. The liraglutide treatments delayed disease onset by two days and decreased the median clinical score from 5 to 2 (p = 0.0003). The neuroprotective nature of liraglutide was investigated in a rat model of middle cerebral artery occlusion (MCAO) [24]. They started with a sample size of 24 rats. Twelve rats received 100μg/kg Liraglutide subcutaneously injected once per day for 1, 3, and 7 days after MCAO surgery, and 12 received a saline vehicle. The liraglutide group saw decreased death of cortical neurons after seven days compared to the vehicle group (p < 0.001). The neuroprotective effects of liraglutide were investigated in a mouse model of cuprizone-induced multiple sclerosis [25]. The study had a sample size of 60 C57B1/6 mice. Thirty mice received oral cuprizone to induce a multiple sclerosis model. Fifteen mice received liraglutide (25 nmol/kg/day i.p.) for four weeks beginning one week post-cuprizone-induced acute demyelination, and 15 received a vehicle. Liraglutide treatment increased brain weight by 15% (p < 0.05). The group treated with liraglutide also had a 35% increase in myelinated nerve fibers compared to the cuprizone control group (p < 0.05).

One study examined the effect of Exendin-4, a GLP-1 agonist, on nerve regeneration post-crush injury [26]. The study had a sample size of 48 Wistar rats divided into four groups; 24 rats were induced with nerve crush injury. Twelve crush injury rats received 2.5 μg/rat/day of Exendin-4 beginning 5 minutes after nerve crush injury, and 12 rats received 0.5 mL of saline vehicle. The sciatic functional index of the crush injury group receiving Exendin-4 returned faster and was significantly higher than the untreated crush injury group (p < 0.01). Another study looked at the effects of GLP-1 agonists on endothelial function in patients with multiple sclerosis [27]. The study had a sample size of 25 patients with multiple sclerosis. 13 patients received 0.75 mg of dulaglutide subcutaneously once per week for 12 months. The control group received no GLP-1 agonist therapy and consisted of 12 patients. After 12 months, the endothelial function of the control group was markedly decreased (2.1 ± 0.5 vs. 1.8 ± 0.6; p = 0.030), while the endothelial function of the GLP-1a group was maintained (2.1 ± 0.6 vs. 2.1 ± 0.7; p = 0.807).

Discussion 

The main objective of these studies is to evaluate the therapeutic potential of GLP-1 agonists in MS management. Various GLP-1 agonists, including NLY01, liraglutide, semaglutide, dulaglutide, and Exendin-4, were investigated. All nine studies demonstrated significant results. In the first study, NLY01 was shown to delay the onset and severity of experimental autoimmune encephalopathy (EAE) symptoms when administered immediately, but the results were not significant when NLY01 was given later [19]. The second study, similar to the first, used liraglutide instead of NLY01 [20]. The use of liraglutide successfully lessened the disease score in EAE mice compared to mice without liraglutide and delayed the onset of EAE by nearly three days. The third study selected semaglutide as the GLP-1 agonist [21]. Similar to the previous studies, semaglutide significantly decreased EAE clinical symptoms. This study also explored semaglutide's effects on demyelination and neurogenesis. cAMP-response element-binding protein (CREB), brain-derived neurotrophic factor (BDNF), and myelin basic protein (MBP) are critical proteins required for neurogenesis, which decreased drastically in EAE mice compared to those given semaglutide. Hence, semaglutide was proven to increase myelination compared to the EAE mice. The fourth study examined dulaglutide as both a prophylactic and a semi-therapeutic against EAE symptoms [22]. Dulaglutide significantly decreased the EAE score as both a prophylactic and a semi-therapeutic, although it was not as effective when used as a semi-therapeutic, but it still improved symptoms. Similarly, the fifth study demonstrates that liraglutide delays clinical disease progression in EAE [23]. The sixth study focused on liraglutide as a neuroprotective agent rather than its effects on EAE symptoms [24]. Liraglutide was shown to improve neurological deficits in motor and sensory functions in mice with MCAO. The seventh study focused on cuprizone-induced MS mice treated with liraglutide [25]. The group treated with liraglutide had overall beneficial results. They were noted to have increased myelinated nerve fibers, leading to a greater brain weight than those mice without liraglutide. The eighth study concerned Exendin-4 treatment following nerve crush injury [26]. The results showed Exendin-4 led to faster and significantly higher improvement in the sciatic functional index, suggesting a positive effect on nerve regeneration. The final study used dulaglutide to treat human MS patients [27]. While endothelial function declined in the control group, it remained steady in the group receiving dulaglutide, suggesting that endothelial dysfunction could play a role in vascular complications, making dulaglutide essential for maintaining endothelial function. These studies contribute greatly to the belief that GLP-1 agonists are a promising treatment for MS in multiple ways.

Many of the studies discussed did not mention safety parameters or any adverse effects of the GLP-1 agonists. The second study noted unexpected deaths in several mice treated with liraglutide, possibly due to the dosage amount given. The fifth study also used liraglutide and stated that it has been proven safe in humans, except for gastrointestinal side effects. Future studies focused on appropriate dosing and human usage should be conducted to ensure GLP-1 agonists are a promising treatment for MS. GLP-1 agonists have also recently been researched as a potential tool for the treatment of other neurodegenerative diseases, such as Parkinson’s disease and Alzheimer’s disease. One study looked at the neuroprotective effects of exenatide on Parkinson’s disease [28]. Exenatide improved the patients’ motor scores, while those given a placebo saw decreased motor scores. It is not certain if exenatide affects the pathophysiology behind Parkinson’s disease, but it is beneficial to those battling the disease. Another study examined the liraglutide-mediated or modulated role and neuroprotective mechanisms in Alzheimer’s disease [29]. This study showed improved cognition in mice by amplifying aerobic glycolysis and decreasing oxidative stress in the brain. Both studies underscore the importance and relevance of GLPs in the treatment of neurodegenerative disorders and the need for more research on the use of GLPs in diseases besides MS.

As research for GLP-1 agonists in the treatment of MS continues, the concern for the cost and availability of these products must be addressed. Some GLPs, such as dulaglutide and semaglutide, have experienced unexpected shortages since early 2023 [30]. This is thought to be caused by the constant increase in demand without adjustment in production. As more and more treatment options with GLP-1 agonists become available, the demand will continue to increase (Table 1).

**Table 1:** Summary of clinical studies, including study model, sample size, drug administration, and effects on MS symptoms. Effects on MS symptoms are expressed using p-values. All p-values < 0.05 are considered statistically significant across all aforementioned studies. MS: Multiple sclerosis.

Author (Year)	Type of Study/Human, Animal, or Lab Model	Sample size(s)	Dose/Administration regimen of GLP-1 Agonist	Effect on MS symptoms (p-value)
Gharagozloo M et al. (2021) [19]	C57BL/6 J and SJL/J Mice	51	NLY01 (10 mg/kg) twice weekly subcutaneous for 42 days	Prevention paradigm delayed the onset and decreased the severity of EAE symptoms (p ≤ 0.0001).
Song S et al. (2022) [20]	Female C57BL/6 Mice (8-10 weeks old)	13-28 per group	Liraglutide 10 μg/kg/day starting from 8 days post immunization	Liraglutide administration decreased the disease score of EAE (EAE vs. EAE + Lira 14.31 ± 2.51 dpi vs. 17.44 ± 4.13 dpi, p = 0.031).
Sadek MA et al. (2023) [21]	Male Swiss-Albino mice (20-25 g)	40	Semaglutide 25 nmol/kg/day i.p.	Semaglutide treatment decreased EAE clinical score (median of 3 to median of 1) compared to the untreated mice (p < 0.05). Semaglutide group had a 60.88% increase in myelinated nerve fibers (p < 0.05).
Chiou HC et al. (2019) [22]	C57BL/6 Mice	29	Dulaglutide prophylactic administration 0, 3, 7, and 10 days post immunization. Semi-therapeutic administration 9, 12, 16, 19, 23, and 26 days post immunization.	Prophylactic dulaglutide treatment decreased EAE clinical score compared to control (3.781 ± 0.2083 to 2.021 ± 0.3053, p < 0.001). Semi-therapeutic treatment decreased clinical score (2.889 ± 0.2887, p < 0.05).
DellaValle B et al. (2016) [23]	Female Lewis Rats	30	Twice daily liraglutide (200 μg/kg s.c.)	Liraglutide treatment delayed disease onset by 2 days and decreased median clinical score from 5 to 2 (p = 0.0003).
Zhu H et al. (2016) [24]	MCAO rats	24	Liraglutide 100μg/kg once per day for 1, 3, and 7 days subcutaneous	Liraglutide decreased death of cortical neurons after 7 days (p < 0.001)
Ammar RA et al. (2022) [25]	Male C57B1/6 Mice (8-10 weeks old, 20-25 g)	60	Liraglutide (25 nmol/kg/day i.p.) for 4 weeks. Introduced at 2nd week of regimen for 4 weeks.	Liraglutide treatment reversed brain weight alterations by 15% (p < 0.05) and increased the number of myelinated nerve fibers by 35% (p < 0.05).
Yamamoto K et al. (2013) [26]	Wistar rats (8 weeks old, 175-210 g)	48	Exendin-4 2.5 μg/rat/day	Exendin-4 increased sciatic functional index post crush injury (p < 0.05).
Hardonova M et al. (2023) [27]	Human study	25	Dulaglutide 0.75mg subcutaneously, once per week, for 12 months	Endothelial function was decreased in the control group (2.1 ± 0.5 vs. 1.8 ± 0.6; p = 0.030) and maintained in the GLP-1a group (2.1 ± 0.6 vs. 2.1 ± 0.7, p = 0.807)

Although there is much discovery ahead regarding the use of GLP-1 agonists, their potential to improve axonal regeneration and remyelination could transform the longevity and quality of life for patients with chronic MS [17]. GLP-1 agonists have increased myelination, delayed clinical manifestations, and improved symptoms as a neuroprotective agent. According to a mouse model study of MS, the GLP-1 agonist NLY01 delayed the onset and decreased the severity of experimental autoimmune encephalomyelitis (p ≤ 0.0001) [19]. Another study suggests that semaglutide resulted in a 60.88% increase in myelinated nerve fibers compared to a control group of EAE (p < 0.05) [21]. Additionally, liraglutide treatments delayed disease onset and decreased the median clinical score from 5 to 2 in animal models (p = 0.0003) [23].

## Conclusions

The neuroprotective effects demonstrated by the significant data results suggest that a variety of GLP-1 agonists could have the capacity to improve the management therapeutics used for the treatment of MS in multiple ways. No clinical trials of GLP-1 agonists for axonal regeneration are currently being conducted. Thus, determining a clearer understanding of the mechanism of action would allow the initiation of large-scale clinical trials needed to determine the potency, safety, and efficacy of these molecules on neurodegenerative disorders. The increase in demand without a corresponding rise in the product's availability creates concern for a potential obstacle as the variety of uses for GLP-1 agonists continues to expand. The future of GLP-1 agonists provides a promising outlook for long-term therapeutic use in MS and other demyelinating disorders.
